# Adverse maternal and fetal outcomes in mouse models of prenatal infections

**DOI:** 10.12688/wellcomeopenres.23556.2

**Published:** 2025-09-09

**Authors:** Evgeniya V Shmeleva, Delia Hawkes, Cecilia Lusuardi, Yasmin Adewusi, Salvatore Valenti, Francesco Colucci

**Affiliations:** 1 Department of Obstetrics and Gynaecology, NIHR Cambridge Biomedical Research Centre, Addenbrooke's Hospital, University of Cambridge School of Clinical Medicine, Cambridge, England, UK; 2 Biology Department, Allen Discovery Center at Tufts, Tufts University, Medford, MA, MA 02155, USA

**Keywords:** prenatal infections, pregnancy, maternal immunity, Toxoplasma gondii, vaccinia virus, immunopathology, mouse models.

## Abstract

**Background:**

Prenatal infections are a leading cause of adverse pregnancy outcomes, yet the mechanisms underlying pathogen-specific effects on maternal and fetal health remain poorly understood.

**Methods:**

Here we conducted a comparative analysis of four mouse models of prenatal infection: *Toxoplasma gondii* (intraperitoneal), vaccinia virus (intranasal), murine cytomegalovirus (intravenous) and influenza A virus (intranasal).

**Results:**

We found markedly different effects on maternal morbidity and mortality, with *T. gondii* causing severe pregnancy-specific pathology leading to maternal mortality by 8 days post-infection, despite similar pathogen loads in pregnant and non-pregnant mice. Vaccinia virus caused prenatal morbidity, while cytomegalovirus and influenza induced only mild, transient effects. The maternal mortality in *T.gondii* infection was most likely due to immunopathology, while vaccinia virus caused prenatal morbidity possibly due to tissue infection. None of the pathogens directly infected the fetuses, yet both *T. gondii* and vaccinia virus significantly impaired both uterine vascular remodelling and fetal growth. Notably, pregnancy was found to be a modifier of local but not systemic immune responses, with reduced inflammatory cytokine production in uterine tissue of infected pregnant mice compared to non-pregnant controls.

**Conclusions:**

These models provide a systematic platform for understanding pathogen-specific mechanisms of pregnancy complications and identifying therapeutic targets.

## Plain language summary

Infections during pregnancy can lead to serious complications for both mother and baby, yet we understand little about why different infections cause varying degrees of harm. This study used mice to compare how four common infections affect pregnancy: the parasite Toxoplasma gondii, vaccinia virus, cytomegalovirus and influenza virus. None of these infections directly reached the developing foetuses, but they affected pregnancy through different mechanisms. In this study we show that the severity of infection during pregnancy does not simply depend on how much the infection spreads, but rather on how the mother's body responded to it. The most dramatic example was Toxoplasma gondii infection, which proved fatal to pregnant mice while causing no obvious illness in non-pregnant mice, despite similar levels of parasites in both groups. Vaccinia virus caused significant but temporary illness in both pregnant and non-pregnant mice. In contrast, cytomegalovirus caused only mild symptoms in mothers but still led to reduced foetal growth, while influenza virus had minimal effects on pregnancy despite its known severity in pregnant women.

## Background

Nearly 20 million children are born underweight every year in the world ^1^. Early onset fetal growth restrictions, which affects a fraction of these children, is associated with worst outcomes. Those with severe fetal growth restriction are exposed to greater risks of both perinatal complications and disease in adulthood ^2– 4^. Perinatal infections with TORCH (toxoplasma, rubella, cytomegalovirus, herpes simplex virus and other pathogens) associate with low birth weight ^5^. While it is known that the severity of congenital infection is due to prenatal infection in utero, there has been no recent progress in the understanding of the basic pathophysiological mechanisms leading to severe outcomes. The stagnation in this field is due to: 1) limited access to human samples for experimentation; 2) limitations and difficulties of animal models; 3) intrinsic complexity of tissue and systemic immune responses.

Besides delivering maternal resources to the fetus, the placenta protects the fetus from potential adverse exposures. Trophoblast cells derived from the fetus, are the cells that form the placenta. They drive, together with maternal immune cells in the decidua, uterine arterial remodelling ^6^. This restructuring of the maternal arterial vasculature in the uterus provides adequate exchanges of nutrients, gases and waste with the developing fetus. Uterine natural killer (uNK) cells are, together with macrophages, the main immune cell populations in the decidua and they are believed to cross talk with fetal trophoblast cells, contributing to vascular remodelling ^7^. The vascular remodelling, which is essential for healthy placentation in both humans and mice, results in dilated, low resistance and high capacitance arteries. Experimental evidence shows that defective placentation and arterial remodelling are associated with adverse outcomes for both mother and child. Preeclampsia, preterm birth and intrauterine growth restriction (IUGR) are frequent pregnancy complications with adverse maternal and neonatal outcomes, as well as future health implications for both mother and offspring ^6^. Prenatal infection may, directly or indirectly, alter vascular remodelling during early pregnancy ^5^.

In this study, we examined four pathogens representing distinct classes of infectious agents with known relevance to pregnancy. Toxoplasma gondii, a protozoan parasite, is a major cause of miscarriage and severe congenital malformations in humans. Vaccinia virus (VACV), a model orthopoxvirus, can induce fetal loss and reproductive pathology in murine models. Murine cytomegalovirus (MCMV) serves as a species-specific analogue of human CMV, which causes intrauterine growth restriction and long-term neurodevelopmental disorders. Influenza A virus (IAV), particularly during pandemic strains, has been linked to increased maternal morbidity and adverse perinatal outcomes. These pathogens differ in tropism, replication kinetics, and immune evasion strategies, allowing for a comparative analysis of pathogen-specific effects on pregnancy.

We aimed here at better determine the systemic and local immunological changes during responses to acute infections during pregnancy. Specifically, we set out to determine whether and how acute infection caused compromised vascular remodelling, defective placentation, and fetal development in models of prenatal toxoplasma, vaccinia virus, cytomegalovirus and influenza virus infection.

## Methods

### Animal procedures

Female C57BL/6 mice aged 7–10 weeks were maintained under specific pathogen-free conditions at the University of Cambridge in accordance with UK Home Office regulations (PPL PP2363781) approved by the Animal Welfare and Ethical Review bodies of the University Biomedical Services of the University of Cambridge, UK. No formal criteria were established a priori for the inclusion or exclusion of animals or data points in this study. All animals that underwent experimental procedures were included in the final analysis. Animals were allocated to control and treatment groups based on a random selection process performed by the researcher. Although no formal computer-generated randomisation sequence was used, the researcher ensured that the allocation was unbiased by selecting animals without any systematic criteria. No specific measures were implemented to randomise the order of treatments or the positioning of cages. All animals were housed under standardised conditions and treated within the same time frame. Mice were acclimatised for at least one week prior to initiating any experimental procedures.

A coding system was implemented throughout the experiment to minimise observer bias. Codes were assigned not only to the group allocation but also to cages, individual mice and all samples. All personnel responsible for administering treatments, conducting measurements, and performing outcome assessments remained blinded to the group allocations as all animals and samples were referred to only by their codes. Data analysis was carried out using the coded identifiers, and the group identities were revealed only after the completion of all analyses. This approach ensured that potential bias was minimised at every stage of the experimental workflow.

All outcome measures were defined prior to the commencement of the experiments. The primary outcome measure was maternal morbidity and mortality. Secondary outcome measures included maternal weight changes, uterine vascular remodelling (quantified by vessel area, lumen area and the vessel-to-lumen ratio in spiral arteries), and fetal and placental weights. In addition, cytokine and chemokine levels in uterine tissue and plasma (including TNFα, IFNγ, IL-6, CXCL10 and IL-1α), as well as immune cell populations in implantation site tissues (characterised by flow cytometry), were assessed. These endpoints were selected to provide a comprehensive evaluation of the impact of prenatal infections on both maternal and fetal health.

For timed matings, females were placed overnight with 3–4-month-old stud males. The presence of vaginal plugs the following morning indicated successful mating and was designated as embryonic day (E) 0.5 ^8^. We plan to study the effects of prenatal infection in both female and male progeny in the future, however in this study we limited the analysis to female mice as we focused on the effect of the prenatal infection on pregnancy.

Pregnant and non-pregnant females were infected with one of four pathogens: *Toxoplasma gondii* (Pru delKu80 delHxgprt strain, 500 parasites, intraperitoneal) at gd 4.5, vaccinia virus (Western Reserve (WR) strain, 2×10 ^3^ PFU, intranasal) at gd 4.5, murine cytomegalovirus (Δm157 strain, 2×10 ^5^ PFU, intravenous) at gd 6.5, or influenza A virus (PR8 strain, 10 ^3^ PFU, intranasal) at gd 4.5. To enable comparison across models, infection time points were selected to align peak immune responses and clinical signs (if any) with mid-gestation (gd 9.5-11.5), when vascular remodelling is actively occurring. T. gondii, VACV and IAV were administered at gd 4.5, which corresponds to peak effects at 5-8 dpi. In contrast, MCMV, which is rapidly cleared from circulation within a few days, was administered at gd 6.5 to ensure its effects also peaked within this critical gestational window. Control animals received matched volumes of vehicle via the same routes. Back titrations were performed for each infection experiment to confirm inoculum doses: *T. gondii* tachyzoites were enumerated by plaque assay on HFF, viral titres were determined by plaque assay on appropriate cell lines (BSC-1 cells for vaccinia virus, MEF for MCMV, and MDCK cells for influenza virus) within 2 hours of preparation.

Body weights were measured daily and compared to initial weights at infection. Animals were culled at various time points depending on the experimental endpoint. At gd 18.5, fetuses and placentas were dissected and weighed individually, with sex noted. Sex was recorded for each fetus, but no consistent sex-specific differences were observed across experimental groups; therefore, sex was not used as a biological variable in subsequent analyses. For earlier timepoints, whole implantation sites were collected. Blood, major organs, implantation sites and placentas were harvested for subsequent analyses.

Mice were euthanised by cervical dislocation, followed by exsanguination to confirm death. To reduce pain, distress, or discomfort in mice, environmental enrichment was provided, such as nesting material, and hiding structures in the cages to promote natural behaviors and reduce stress. Housing conditions were optimised to allow for natural behaviors. Gentle handling techniques were used. Both researchers and technicians were properly trained in animal handling and pain assessment techniques. Mice were regularly observed for signs of distress like decreased activity, hunched posture, piloerection, or changes in eating/drinking patterns. Humane endpoints were implemented as indicated below. Mice received an administration of a category 1, 2 pathogen or control vehicle. Animals were closely monitored. The lowest dose of pathogens capable of inducing the clinical symptoms required for the study was used. The route of administration was selected to minimize distress while ensuring the clinical symptoms required for the study were induced. Pregnant mice were humanely killed before they gave birth at day 18.5 of gestation (day of vaginal plug discovery = 0.5 day of gestation). Weight loss was monitored and when it reached more than 15%, mice were killed. If the adverse effects of infection appeared greater than mild, including if mice appear hunched, or display other non-transient moderate clinical signs, e.g. piloerection, subdued behaviour, diarrhoea, they were killed by schedule 1 method. Any animal that losed 15% of its body weight gradually when compared to age-matched controls was killed.

### Pathogen load quantification

For *T. gondii* quantification, uterine tissues were collected at 7 days post-infection, homogenised in sterile PBS (0.4 ml) using a bead beater (4 cycles of 20 seconds), and DNA was extracted using DNeasy UltraClean Microbial Kit (Qiagen). Parasite burden was determined by qPCR targeting the *T. gondii* GRA2 gene.

For VACV quantification, uterine tissues and lungs were collected at 7 days post-infection, homogenised in sterile PBS (0.4 ml) using a bead beater (4 cycles of 20 seconds for uterine tissue, 2 cycles for lungs). Homogenates underwent three freeze-thaw-sonication cycles and were titrated by plaque assay on BSC-1 cells. Viral titres were calculated as plaque-forming units (PFU) per gram of tissue.

For IAV quantification, uterine tissues and lungs were collected at 7 days post-infection, homogenised in sterile PBS (0.4 ml) using a bead beater (4 cycles of 20 seconds for uterine tissue, 2 cycles for lungs). Homogenates were clarified by centrifugation (10,000 × g, 20 minutes, 4°C), and viral titres were determined by plaque assay on MDCK cells. Viral titres were calculated as plaque-forming units (PFU) per gram of tissue.

### Vascular remodelling analysis

Maternal vascular remodelling during pregnancy was assessed as previously described in ref. 9. Briefly, gd 9.5 implantation sites, together with uterine horns, were collected, fixed in 10% neutral buffered formalin for 6 hours, and embedded in paraffin. Sections (7 µm) were stained with haematoxylin and eosin following standard protocols. For quantification of vascular remodelling, vessel and lumen areas were measured in cross-sections of spiral arteries in the decidua basalis using ImageJ software (NIH, https://imagej.net/ij/ Five largest and roundest vessels within each implantation site were assessed. For each vessel, the lumen area and total vessel size were measured, with the ratio of total vessel size to lumen area used as an indicator of remodelling status. To ensure reproducibility, measurements were performed in triplicate at each implantation site, resulting in 15 data points per site. The mean of these 15 measurements was calculated to determine the average size of the largest vessels. A minimum of three implantation sites examined per animal. The vessel-to-lumen ratio was calculated by dividing the total vessel area (including the vessel wall) by the lumen area. All measurements were performed blind to the experimental group. Spiral artery remodelling was assessed in T. gondii, VACV and MCMV models; IAV-infected tissues were not included in this analysis due to the absence of overt pathology.

### Pathogen localisation analysis

For pathogen distribution analysis, implantation sites were collected from mice infected with either mCherry-expressing *T. gondii* (Pru delKu80 delUPRT mCherry) or TagRFP-T-expressing vaccinia virus (WR strain). Implantation sites were collected at 5 and 7 days post-infection, fixed in 4% paraformaldehyde for 4 hours at 4°C, embedded in OCT compound and cryopreserved. Cryosections were mounted using ProLong Diamond Antifade mountant with DAPI (ThermoFisher, P36966) and imaged using a Leica LSM700 confocal microscope. Multiple sections from at least three implantation sites per animal were examined, with a minimum of 4 animals per group. Fluorescent signal intensity and distribution were analysed across decidual and placental regions, with particular attention to the maternal-fetal interface.

### Cytokine and chemokine analysis

Uterine tissues and blood were collected at gd 11.5. For tissue analysis, implantation sites were homogenised in sterile PBS (0.4 ml) using a bead beater (4 cycles of 20 seconds), centrifuged at 10,000 × g for 20 minutes at 4°C, and supernatants were collected. Blood was collected into K3 EDTA tubes and centrifuged at 1,000 × g for 15 minutes at 4°C to obtain plasma. Luminex assay kits were used to measure plasma and uterine tissue levels of CXCL10, IL-1α, IL-6, IFN-γ, and TNF-α. Assays were performed according to manufacturer's instructions and analysed using Luminex MAGPIX system with xPONENT software (Luminex Corporation). Results are expressed as pg/ml for plasma and pg/g tissue for uterine samples.

### Flow cytometry analysis of uterine leukocytes

Uterine/implantation site leukocytes were isolated at gd 11.5 (7 days post-infection) following previously described protocols ^8^. To distinguish tissue-resident from circulating populations, blood cells were labelled in vivo by intravenous injection of anti-CD45-AF488 antibody (clone 30-F11, BioLegend, #103122; 3 µg per mouse) 3 minutes before tissue collection.

Single-cell suspensions were stained with Zombie NIR viability dye (BioLegend, #423105) and blocked with TruStain FcX (BioLegend, #101320) before staining with fluorochrome-conjugated antibodies: CD45 (clone 30-F11, BD, #557235, PerCP), CD3 (clone 145-2C11, BioLegend, #100351, BV605), CD4 (clone GK1.5, BD, #563790, BUV395), CD8 (clone 53-6.7, BD, #612898, BUV805), NK1.1 (clone PK136, BD, #564143, BV650), NKp46 (clone 29A1.4, BioLegend, #137637, BV785), CD49a (clone Ha31/8, BD, #562113, AF647), CD11b (clone M1/70, BD, #562605, BV421), and Ly6G (clone 1A8, BioLegend, #127633, BV510). For intracellular staining, cells were fixed and permeabilised using Foxp3 Transcription Factor Staining Buffer Set (eBioscience, #00-5523-00) and stained for Eomes (clone Dan11mag, eBioscience, #12-4875-82, PE).

Data were acquired on a Cytek Aurora spectral cytometer (Cytek Biosciences) and analysed using FCS Express software (De Novo Software). Flow cytometry data analysis can alternatively be performed using Flowing Software 2 ( http://www.flowingsoftware.com/) or the R package ‘flowCore’ via Bioconductor ( https://www.bioconductor.org/packages/release/bioc/html/flowCore.html). Cell populations were defined as: NK cells (CD45+CD3-NK1.1+NKp46+) subdivided into conventional NK cells (CD49a-), tissue-resident NK cells (CD49a+Eomes+), and ILC1s (CD49a+Eomes-); T cells (CD45+NK1.1-NKp46-CD3+) further categorised into CD8+ T cells (CD4-CD8+), CD4+ T cells (CD4+CD8-), double-negative T cells (CD4-CD8-), and double-positive T cells (CD4+CD8+); neutrophils (CD45+CD3-NKp46-CD11b+Ly6G+), and monocytes/macrophages/dendritic cells (CD45+CD3-NKp46-CD11b+Ly6G-) (Extended data, Figure E1). Absolute cell numbers were calculated per gram of tissue.

For confirmation of origin of *T. gondii*-infected cells, implantation sites were collected from mice infected with mCherry-expressing *T. gondii* (Pru delKu80 delUPRT mCherry). Leucocytes were isolated at 7 days post-infection as described in the flow cytometry analysis section, including intravital CD45-AF488 labelling. Single-cell suspensions were stained with CD45-PerCP and Ly6G-BV510 antibodies and fixed with 8% paraformaldehyde. Ly6G-positive and Ly6G-negative populations were sorted using a BD Influx cell sorter, with 8,000 cells per population deposited directly onto microscope slides. Cells were mounted using ProLong Diamond Antifade mountant with DAPI (ThermoFisher, P36966) and cured overnight at 4°C in the dark. Images were acquired using a Leica LSM700 confocal microscope to visualise DAPI nuclear staining and mCherry signal from *T. gondii*. Multiple fields were examined from samples collected from four mice to assess parasite localisation within neutrophils.

### Statistical analysis

Statistical analyses were performed using SPSS (v.29, IBM, https://www.ibm.com/analytics/spss-statistics-software) and GraphPad Prism (v.10.4.0, https://www.graphpad.com/scientific-software/prism/). Statistical analyses can alternatively be performed using R ( https://www.r-project.org/). To verify that the data met the assumptions underlying the statistical tests, we initially assessed the normality of each dataset using the Shapiro-Wilk test and by visual inspection of Q-Q plots. Non-parametric Mann-Whitney U tests were used for comparisons between two experimental groups. For longitudinal analyses of weight changes, two-way repeated measures analysis of variance (ANOVA) was applied, with time and experimental condition as factors. P values < 0.05 were considered statistically significant; *p < 0.05, **p < 0.01, ***p < 0.001, ****p < 0.0001. Effect sizes with corresponding confidence intervals were not computed for the analyses presented in this study.

## Results

### Morbidity, mortality and tissue pathology upon prenatal infections

**Figure 1.**
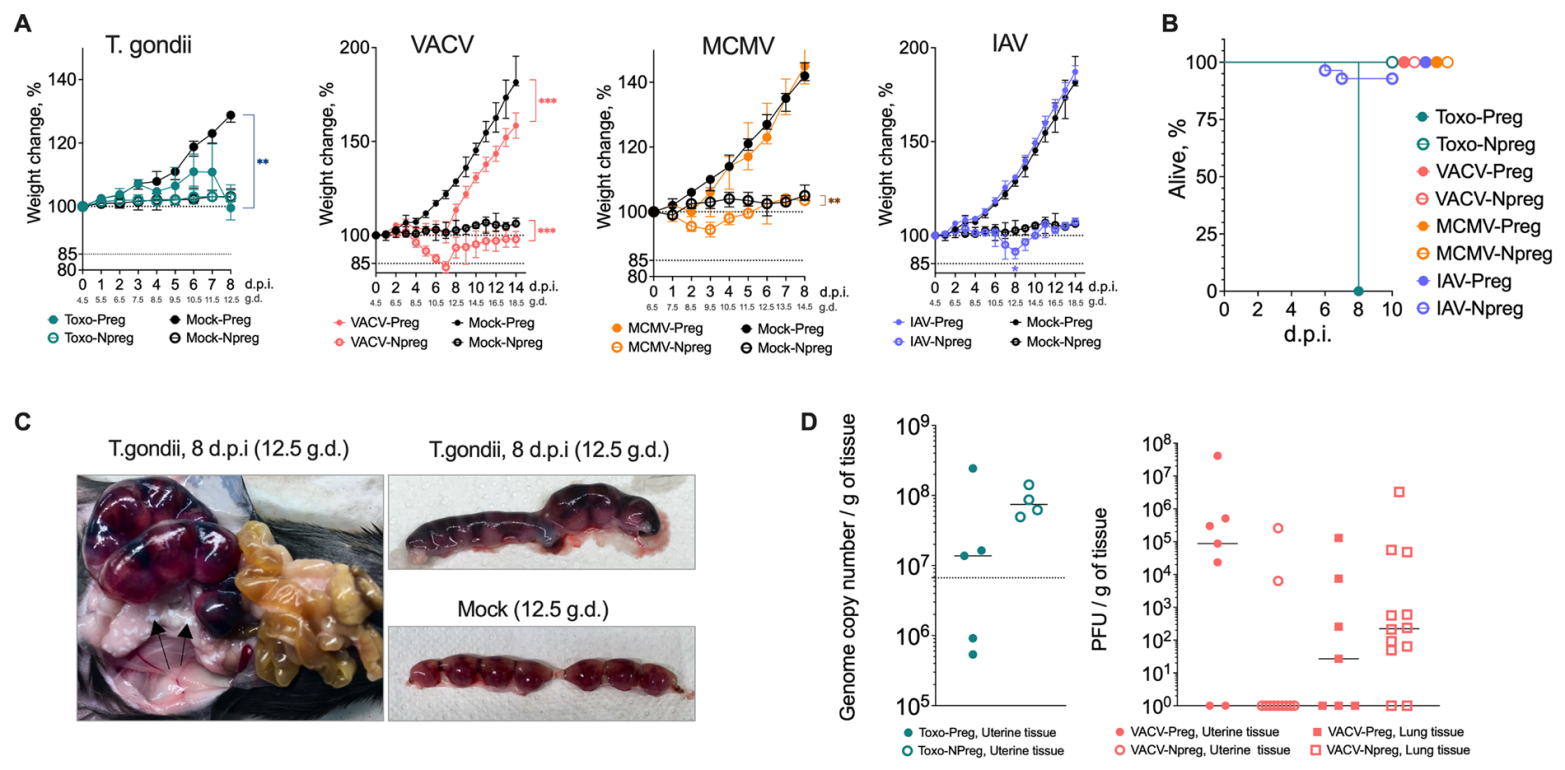
Pathogen-specific effects on maternal health and mortality during pregnancy. **A**) Weight plots for infected and mock-infected pregnant and non-pregnant mice. *T. gondii*-infected pregnant mice showed impaired weight gain in pregnant mice and eventual death, while non-pregnant infected mice maintained weight. VACV infection caused sustained weight loss in both pregnant and non-pregnant mice, with incomplete recovery by 14 dpi. Initially, both pregnant and non-pregnant mice infected with MCMV experienced minor weight loss but all animals recovered by 6dpi. IAV-infected pregnant mice maintained normal pregnancy-associated weight gain, while some non-pregnant IAV-infected mice showed significant weight loss. Values are presented as percentage change from initial weight (100%), with gestational days (g.d.) and days post-infection (d.p.i.) indicated. Medians and interquartile ranges are shown, n = 4 -12 mice per group. The experiments were performed three times, and representative data from one experiment are presented. **B**) Survival curves showing 100% mortality in *T. gondii*-infected pregnant mice by 8 dpi, while non-pregnant infected mice survived. Some mortality was observed in non-pregnant IAV-infected mice between 6-8 dpi, while all other groups survived until planned endpoints. N = 4 -12 mice per group. The experiments were performed three times, and representative data from one experiment are presented. **C**) Representative images of implantation sites at 8 dpi (12.5 g.d.) showing vascular congestion in *T. gondii*-infected tissue compared to mock-infected controls. **D**) Pathogen loads in uterine tissue showing *T. gondii* genome copy numbers (at 7 dpi) between pregnant and non-pregnant mice. VACV loads (at 7 dpi) were detected more frequently in the uterus of pregnant mice compared to non-pregnant mice, while comparable viral loads were found in the lung. Medians and individual values are shown, n = 4 -12 mice per group. The dashed line represents the assay’s lower limit of detection. The experiments were performed twice and representative data from one experiment are shown.

Intraperitoneal infection with *Toxoplasma gondii* at gestation day (gd) 4.5 resulted in the most severe morbidity and mortality in pregnant mice but caused no obvious signs of pathology in non-pregnant mice. Both toxoplasma-infected and mock-infected pregnant mice gained weight due to the progression of pregnancy; however, the weight gain in toxoplasma-infected mice was delayed, and they even lost weight at 4 days post-infection (dpi) ( Figure 1A). The weight loss in toxoplasma-infected pregnant mice correlated with a steep decline in health observed at 6 dpi. By 8 dpi, all toxoplasma-infected pregnant mice had to be humanely culled because of poor health, characterised by oily coats, fur ruffling, trembling and arched backs ( Figure 1B). In comparison, the non-pregnant toxoplasma-infected mice sustained no weight loss, and no clinical signs were observed throughout the eight days ( Figure 1B). Dissection of the toxoplasma-infected and mock-infected implantation sites demonstrated no notable differences or signs of inflammation before 7 dpi. However, the of the toxoplasma-infected implantation sites at 7 and 8 dpi showed a dramatic change and were observed to be stiff with vascular congestion ( Figure 1C). Interestingly, despite the severe clinical signs caused by toxoplasma infection in pregnant mice compared to non-pregnant mice, there was no significant difference in pathogen load in uterine tissue between the two of the toxoplasma-infected groups ( Figure 1D).

Intranasal infection with Vaccinia Virus (VACV) at gd 4.5 did not cause any clinical signs other than weight loss in both pregnant and non-pregnant VACV-infected mice, starting at 4 dpi ( Figure 1A). The weight loss in VACV-infected mice was not as pronounced as that caused by toxoplasma infection; however, it was sustained, and VACV-infected mice did not fully recover until the end-point at 14 dpi. Upon dissection of implantation site tissues from VACV-infected mice, no discernible macroscopic difference was detected between the different groups. The pathogen load was greater in the uterus of 5/7 pregnant VACV-infected mice but was detected only in 2/11 VACV-infected non-pregnant mice. However, pathogen loads were similar in the lungs of both pregnant and non-pregnant VACV-infected mice ( Figure 1D). In two independent experiments, plaque assays performed on ovary homogenates at 7 dpi detected no infectious virus (n = 10, detection limit ≈ 50 PFU/g), suggesting that VACV did not disseminate to ovarian tissue under our experimental conditions (data not shown).

Intravenously infection with murine cytomegalovirus (MCMV) at gd 6.5 in both pregnant and non-pregnant mice caused mild, transient weight loss, followed by recovery to weights similar of mock-infected animals ( Figure 1A). Neither group experienced any other clinical symptoms. To confirm successful infection and assess viral dissemination, we quantified MCMV titres in pregnant mice at 3 dpi (gd 9.5). Infectious virus was detected in the uterus, spleen, and liver, but not in blood (Fig. E 2B).

Intranasal infection with influenza virus (IAV) at gd 4.5 caused mild symptoms with a full recovery. These symptoms included reduced activity and oily coats around 7-8 dpi. The weight gain associated with pregnancy progression was similar in the IAV-infected and mock-infected mice ( Figure 1A). However, some of the non-pregnant IAV-infected mice had to be culled due to significant weight loss between 6 and 8 dpi ( Figure 1B). IAV was undetected in the uterine tissue of either pregnant or non-pregnant IAV-infected mice, while viral loads were similar in the lungs of the two groups (Fig. E 2A).

### Effect of prenatal infections on uterine vascular remodelling and fetal growth

**Figure 2.**
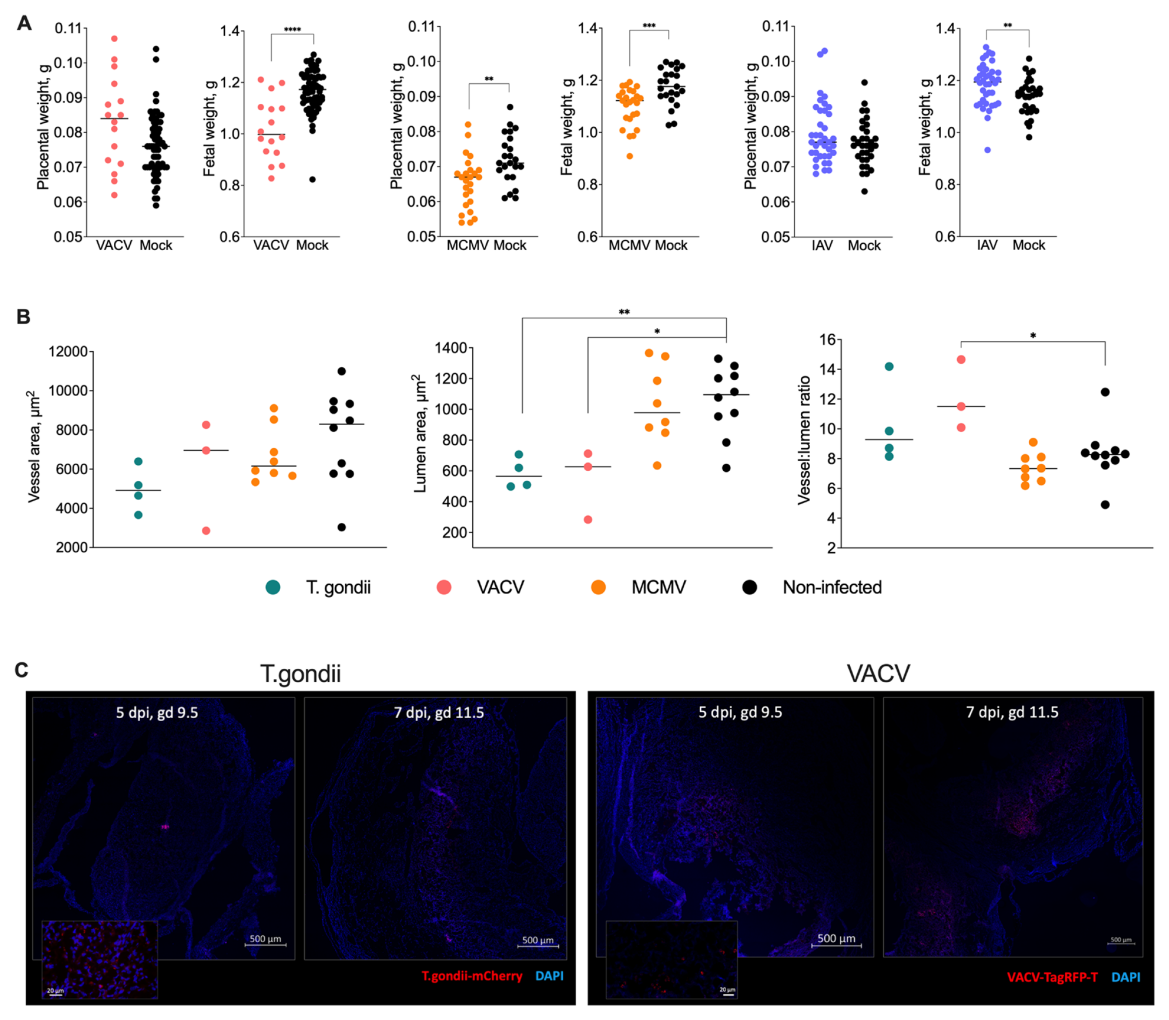
Infections during pregnancy impair vascular remodelling and fetal growth. **A**) Fetal and placental weights at gd 18.5. VACV and MCMV infection reduced fetal weights, whilst IAV infection resulted in increased fetal weights compared to mock-infected controls. Only MCMV infection significantly reduced placental weights. Medians and individual values are shown, n = 16 -68 fetus or placentas per group. The experiments were performed three times, and representative data from one experiment are presented. **B**) Quantification of maternal spiral artery remodelling at gd 9.5. Both *T. gondii* and VACV infection significantly reduced lumen areas, with VACV infection also increasing vessel:lumen ratios, indicating impaired vascular remodelling. MCMV infection did not affect vascular parameters. Data points represent individual implantation sites, with each point reflecting the average of 15 measurements (see Methods). Medians and individual values are shown, n = 4 -12 mice per group. The experiments were performed at least twice, and representative data from one experiment are presented. **C**) Representative confocal microscopy images showing distribution of mCherry-expressing *T. gondii* (left panels) and TagRFP-T-expressing VACV (right panels) in implantation site sections at 5 and 7 days post-infection. *T. gondii* localised primarily in decidual regions, while VACV distributed throughout decidual and placental regions, with stronger signal at 7 days post-infection. Neither pathogen was detected in embryonic tissue.

Fetal weight at term was significantly lower in VACV- and MCMV-infected mice, while it was surprisingly higher in IAV-infected animals. Placental weights were reduced only in MCMV-infected mice ( Figure 2A). Litter sizes did not differ between infected and mock-infected groups. Because prenatal toxoplasma infection caused severe morbidity, the pregnant toxoplasma-infected mice had to be culled, so we could not determine the effect of toxoplasma infection on fetal or placental weight at term.

To determine the effect of prenatal infections on uterine vascular remodelling, we measured the area of whole vessels and their lumens, and the ratio between the two in mice infected with toxoplasma, VACV and MCMV, at mid-gestation. The lumen area was significantly reduced by toxoplasma and VACV infection and the vessel-to-lumen ratio was significantly greater in VACV-infected tissues ( Figure 2B). MCMV did not appear to have any pronounced effect on the vasculature. To localise the infection across the implantation sites, we used mCherry-tagged *Toxoplasma gondii* and TagRFP-T-tagged VACV ( Figure 2C). We observed disseminated infection throughout the decidua and trophoblast ( Figure 2C), with increasing intensity from 5 dpi to 7 dpi. While both toxoplasma and VACV were detected in the uterine tissue, including the trophoblast, there was no signal of either pathogen in the embryos.

### Tissue and systemic immune responses to perinatal infections

**Figure 3.**
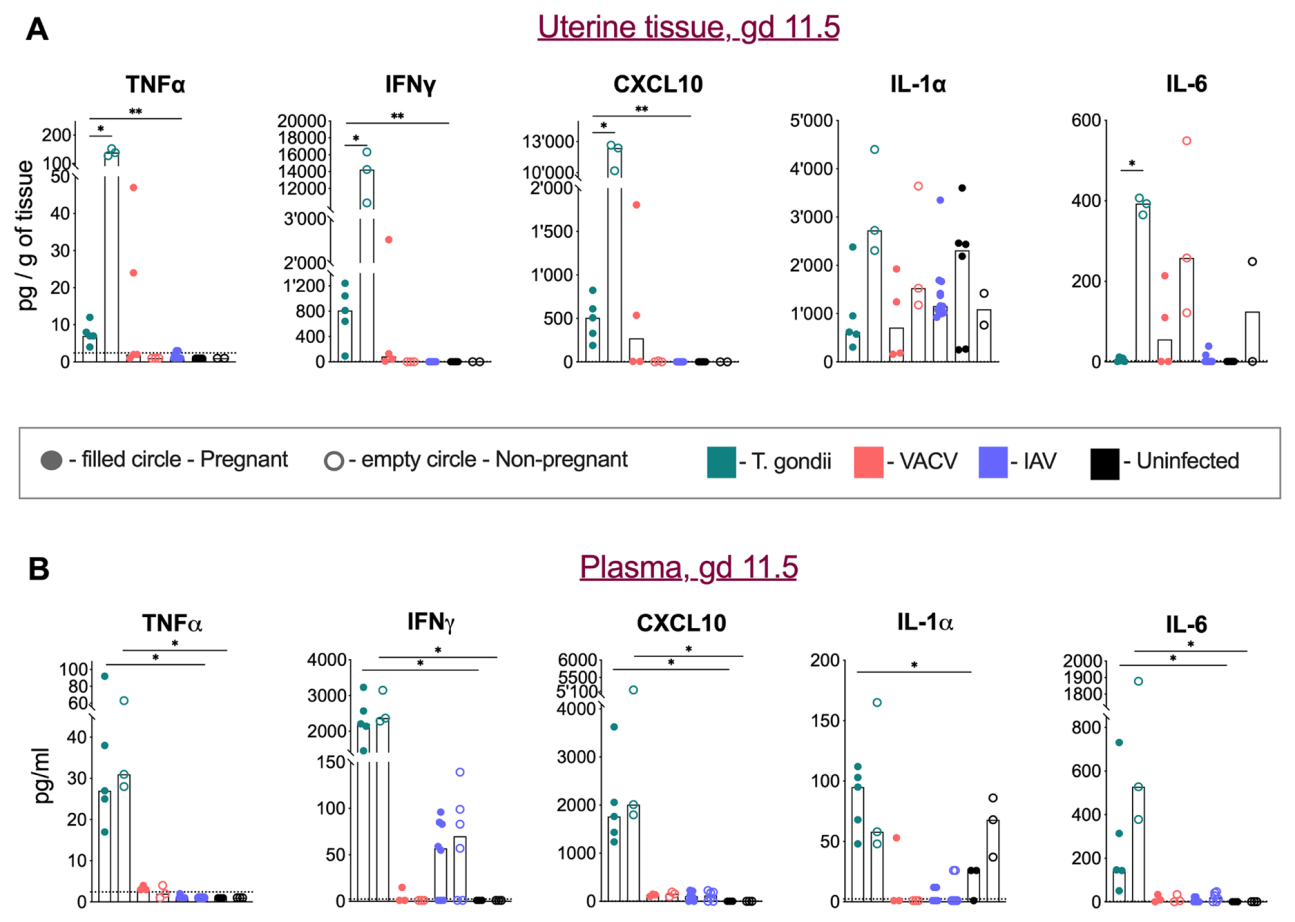
Tissue and systemic cytokine responses in infection during pregnancy. **A**) Cytokine and chemokine levels in uterine tissue homogenates at gd 11.5. *T. gondii* infection (turquoise colour) induced significant increases in TNFα, IFNγ, CXCL10 and IL-6 levels in pregnant mice (filled circles) compared to non-infected controls (empty circles). This inflammatory response was attenuated in pregnant infected mice. VACV infection resulted in elevated IL-6 levels in some animals. IL-1α showed distinct regulation, with higher baseline levels in healthy pregnancy, which decreased during both *T. gondii* and VACV infections. Medians and individual values are shown, n = 2 -12 mice per group. The experiment was performed once. **B**) Plasma cytokine and chemokine concentrations at gd 11.5. *T. gondii* infection (turquoise colour) induced systemic elevation of TNFα, IFNγ, CXCL10, IL-1α and IL-6, with comparable levels between pregnant and non-pregnant mice. VACV infection did not significantly alter plasma cytokine levels, while IAV infection tended to specifically increase IFNγ levels. Medians and individual values are shown, n = 2 -12 mice per group. The experiment was performed once.

We determined the tissue and serum levels of TNF-α, IFN-γ, CXCL10, IL-1α and IL-6 in pregnant and non-pregnant mice infected with toxoplasma, VACV, IAV and in mock-infected mice at gd 11.5. The greatest change in uterine tissue was the significant rise in TNF-α, IFN-γ, CXCL10, and IL-6 ( Figure 3A), upon T. gondii infection, suggesting that the intraperitoneal infection caused acute inflammation in the uterus. However, the tissue of pregnant mice displayed a much-reduced rise in TNF-α, IFN-γ, and CXCL10, and no rise in IL-6, suggesting that pregnancy blunts the local inflammatory response. Intranasal VACV infections also caused a local rise of IL-6. Analysis of IL-1α in uterine tissue revealed a distinct pattern from other measured cytokines. In uninfected controls, pregnant mice showed higher IL-1α levels compared to non-pregnant group. However, this pattern was reversed in both Toxoplasma and VACV infections, where pregnant mice displayed lower IL-1α levels compared to infected non-pregnant animals.

Systemically, we observed increased levels of TNF-α, IFN-γ, CXCL10, IL-1α and IL-6 in toxoplasma-infected mice, no elevated levels in VACV-infected mice, and high levels of IFN-γ in IAV-infected mice. However, there were no significant differences in systemic responses between pregnant and non-pregnant mice ( Figure 3B), suggesting that pregnancy affects local but not systemic immune responses.

**Figure 4.**
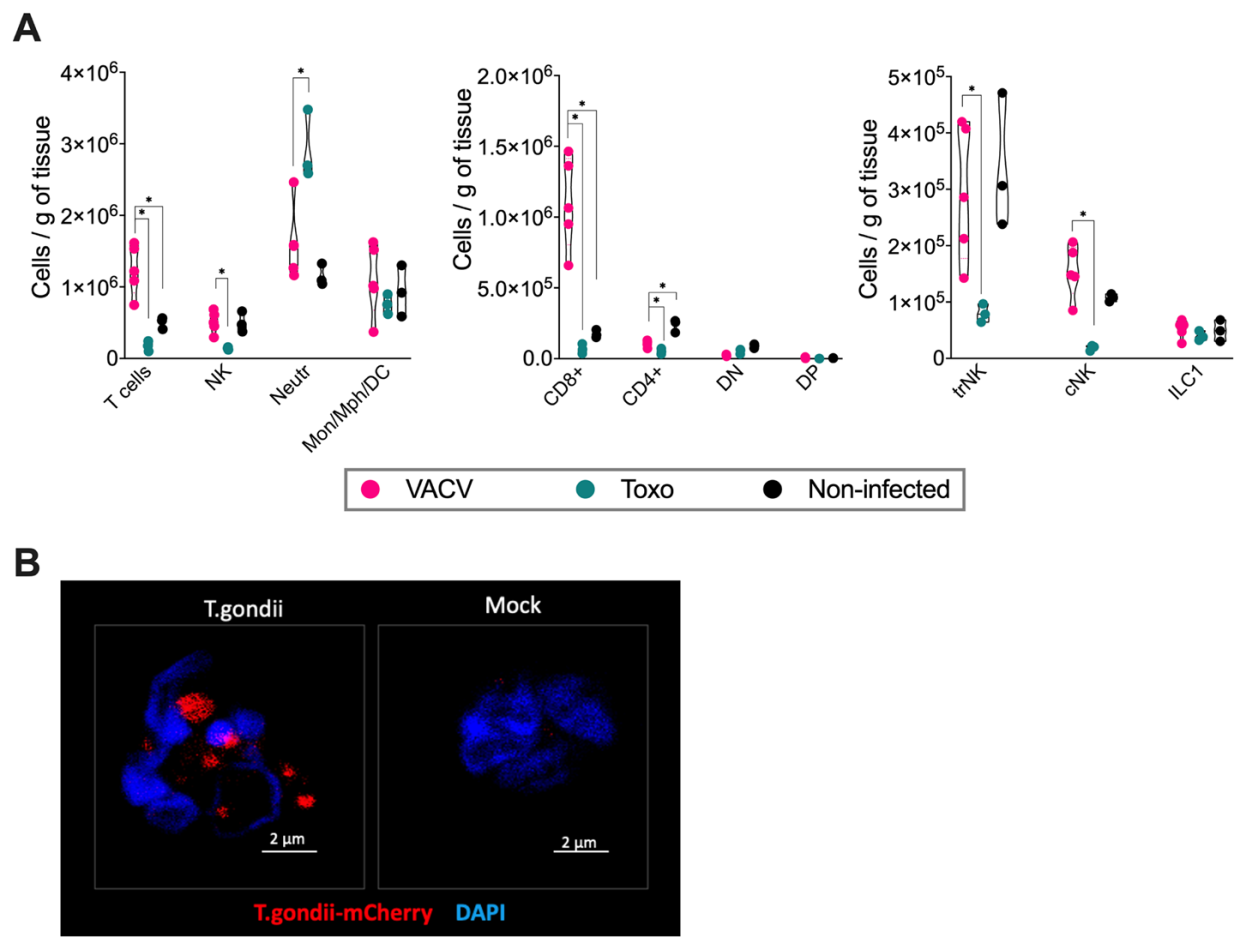
Differential immune cell responses to *T. gondii* and VACV infection during pregnancy. **A**) Absolute numbers of immune cell populations in implantation site tissue at gd 11.5 (7 dpi). *T. gondii* infection resulted in significant reduction of tissue-resident NK (trNK) and conventional NK (cNK) cells, CD8+ and CD4+ T cells, but markedly increased neutrophil numbers in pregnant mice compared to pregnant mock controls. VACV infection led to increased CD8+ T cells and decreased CD4+ T cells, with no significant changes in other examined populations. “DP” - double-positive (CD4+CD8+) T cells; “DN” - double-negative (CD4-CD8-) T cells. N = 3 -5 mice per group. Data show violin plots with individual values from one representative experiment of two independent repeats. **B**) Confocal microscopy of sorted Ly6G+ cells from implantation sites of mice infected with mCherry-expressing *T. gondii* at 7 dpi. The segmented nuclei of Ly6G+ cells confirm their identity as neutrophils. The microscopy images demonstrate that these neutrophils are infected by *T. gondii*.

We carried out an analysis of uterine/implantation site tissues of both toxoplasma- and VACV-infected mice, comparing them to mock-infected controls. Pregnant mice infected with toxoplasma showed reduced NK, CD8 ^+^ and CD4 ^+^ T cell counts while neutrophils were increased substantially in comparison with mock-infected animals ( Figure 4A). The reduction in total NK cells was primarily driven by a significant decrease in tissue-resident NK (trNK) and conventional NK cells (cNK) in toxoplasma-infected pregnant mice compared to uninfected pregnant controls, while ILC1 numbers remained relatively stable. Using mCherry-tagged *Toxoplasma gondii*, we were able to locate toxoplasma within the neutrophils ( Figure 4B). Focal infection points were found in the decidua at 5 dpi. This decreased slightly by 7 dpi but remained detectable.

Analysis of implantation sites from VACV-infected group revealed significant increases in CD8 ^+^ T cells and decline in CD4 ^+^ T cells compared to non-infected controls ( Figure 4A). There were no significant changes in other examined immune cell populations.

## Discussion

This study presents a comparison of four mouse models of prenatal infections - *Toxoplasma gondii*, Vaccinia Virus, Influenza Virus and Cytomegalovirus - revealing distinct pathogen-specific effects on maternal health, fetal development and immune responses. None of the pathogens caused direct fetal infection. However, findings demonstrate that pregnancy fundamentally alters host responses to infection. The most dramatic effects were observed in *T. gondii* infection, where pregnancy transforms an otherwise well-tolerated infection into a lethal condition. Vaccinia virus caused moderate morbidity, while influenza and cytomegalovirus resulted in only mild and transient clinical signs.

While *Toxoplasma gondii* infection in humans is generally asymptomatic, experimental infection in mice may be severe and even fatal, depending on the parasite strain or its stage, the route of administration and, during pregnancy, the gestational age. Prenatal *Toxoplasma gondii* infection in both humans and mice causes pregnancy loss and, in humans, severe congenital toxoplasmosis which affects brain, eyes, and ears. The earlier the infection occurs during human pregnancy, the more severe the clinical presentations are likely to be. We modelled *Toxoplasma gondii* infection in early pregnancy in mice and observed severe morbidity and mortality about a week after infection ( Figure 1B). This was not due to intrinsic pathogenicity of the strain used, nor to the pathogen load or the route of administration, because non-pregnant toxoplasma-infected mice experienced no weight loss ( Figure 1A) or clinical signs, despite the pathogen load detected in the uterine tissue being equivalent in pregnant and non-pregnant mice ( Figure 1D). The pathogenicity of the infection was due to tissue damage caused by local inflammation at the implantation site. However, the tissue levels of inflammatory cytokines were less elevated during pregnancy ( Figure 3A), suggesting two conclusions. Firstly, pregnancy may blunt the acute inflammation in the uterus. Secondly, despite the inflammation was milder in the uterus, it still was not tolerated and this lead to both pregnancy loss and systemic illness. Of note, systemic illness was not observed in the non-pregnant toxoplasma-infected mice. The cellular components of the inflammation demonstrated acute tissue inflammation with presence of abundant neutrophils, which were infected with the parasite ( Figure 4). The presence of parasites within neutrophils, confirmed by our imaging of sorted Ly6G+ cells, suggests these cells may serve as vehicles for parasite dissemination ( Figure 4B). We also detected a reduced number of lymphocytes, particularly NK and T cells ( Figure 4A). Previous studies have shown that IFN-γ production by NK cells can both protect against Toxoplasma infection and cause immunopathology during pregnancy. The vascular pathology we observed in *T. gondii* infection, characterised by reduced vessel lumen area and evidence of congestion ( Figure 2B), provides a potential mechanistic link between immune dysregulation and adverse outcomes. The timing of clinical deterioration (6–7 days post-infection) coincides with when adaptive immune responses typically develop, suggesting that inappropriate adaptive responses may trigger vascular dysfunction. The localisation of parasites primarily in the decidua, rather than in the placenta or fetus, indicates that direct parasite invasion is not necessary for inducing pathology. Instead, our results showed that uterine inflammation without fetal infection is the reason for pregnancy loss upon early prenatal infection with *Toxoplasma gondii*.

Vaccinia virus infection during pregnancy presents a distinct pattern of pathogenesis compared to toxoplasma infection, causing significant morbidity but not mortality. Despite causing pronounced weight loss in both pregnant and non-pregnant mice, VACV-infected pregnant mice showed eventual recovery, suggesting that pregnancy does not exacerbate the overall severity of VACV infection ( Figure 1A, B). However, our data reveal that pregnancy alters local immune responses with potential implications for fetal development. A notable finding was the increased detection of VACV in uterine tissue of pregnant compared to non-pregnant mice, despite similar viral loads in the lungs ( Figure 1D). This pregnancy-specific tropism suggests that gestational changes in the uterine environment may favour viral replication or persistence. The impaired vascular remodelling in VACV-infected pregnant mice, evidenced by reduced lumen areas and increased vessel-to-lumen ratios, suggests that viral infection disrupts crucial pregnancy-specific vascular adaptations ( Figure 2B). This vascular dysfunction may contribute to the observed reduction in fetal weight ( Figure 2A), even in the absence of direct fetal infection ( Figure 2C). The immune response to VACV infection during pregnancy was marked by a significant expansion of CD8+ T cells and concurrent reduction in CD4+ T cells, consistent with a typical antiviral response ( Figure 4A). However, unlike toxoplasma infection, VACV did not induce substantial changes in NK cell populations or trigger massive neutrophil infiltration ( Figure 4A). Moreover, while VACV infection induced elevated IL-6 levels in uterine tissue, it did not trigger the broad inflammatory cytokine response observed with toxoplasma infection ( Figure 3). This more contained inflammatory response, particularly in pregnant animals, suggests that pregnancy-associated immune modulation may help limit immunopathology during viral infection, albeit at the cost of potentially reduced viral clearance in reproductive tissues.

Earlier work using high-dose VACV in BALB/c dams reported extensive viral dissemination, including infection of the ovaries and pregnancy loss ^10^. In that study, BALB/c mice were infected intravenously or intraperitoneally with 2 × 10 ^6^ PFU WR VACV at mid-gestation (gd 11–12). These effects were not observed in our model, likely due to differences in mouse strain, infection dose, and gestational timing. BALB/c mice are known to be more susceptible to vaccinia virus compared to C57BL/6 mice ^11, 12^. Our study used C57BL/6 mice and a 1000-fold lower intranasal dose (2 × 10 ^3^ PFU), administered at gd 4.5. Under these conditions, we did not detect virus in the ovaries by plaque assay.

In humans, prenatal influenza infection causes more severe symptoms and higher mortality rates. Contrary to pregnant humans, it has been shown that pregnant mice exhibit enhanced immune protection against severe flu infections, particularly in their nasal cavities ^13^. Our data support this observation, showing that pregnant mice maintained normal weight gain and experienced only mild, transient symptoms ( Figure 1A, B). Several murine studies, particularly those employing BALB/c mice, late-gestation infection or highly pathogenic or high-dose influenza strains, have reported pregnancy-specific morbidity and adverse fetal outcomes ^14– 16^. Conversely, recent work in C57BL/6 animals has demonstrated pregnancy-associated protection mediated by augmented nasal antiviral immunity ^13^. Our early-gestation, low-dose PR8 model therefore represents one end of this spectrum, highlighting that the impact of IAV on pregnancy is heavily influenced by host genetics, viral factors and inoculation parameters. Interestingly, some non-pregnant mice exhibited significant morbidity requiring euthanasia ( Figure 1B), even though viral loads were similar in the lungs of the two groups (Fig. E2A) and the plasma cytokine levels were comparable ( Figure 3B). The increased fetal weights observed in IAV-infected dams ( Figure 2A), while unexpected, might result from altered maternal metabolism or compensatory growth mechanisms triggered by the infection. However, this observation requires further investigation to understand the underlying mechanisms.

Human cytomegalovirus (HCMV) remains a leading cause of congenital infections causing severe developmental abnormalities and long-term sequelae. While HCMV and MCMV differ in their species specificity, both share similar cellular tropism and capacity to establish persistent infection. According to our results, while infected dams showed only transient weight loss with rapid recovery ( Figure 1A), both fetal and placental weights were significantly reduced at term ( Figure 2A), which suggests that MCMV infection disrupts placental function without triggering overt systemic inflammation. Unlike toxoplasma and VACV infections, MCMV did not impair maternal vascular remodelling at mid-gestation ( Figure 1B), indicating that the observed fetal growth restriction likely stems from other mechanisms. Previous studies have reported severe pregnancy outcomes following MCMV infection, including fetal growth restriction, placental inflammation, and impaired vascular remodelling ^17– 19^. In our study, C57BL/6 mice were infected intravenously at gd 6.5 with the Δm157 MCMV strain. This particular combination of viral strain, host background, infection route and timing may account for the milder phenotype we observed compared to previous reports ^19– 21^. Whereas previous work demonstrated that corpus luteum infection and subsequent progesterone insufficiency caused pregnancy loss in BALB/c mice infected with wild-type MCMV ^19^, we hypothesise that the absence of severe pregnancy outcomes in our model reflects more subtle, strain- and virus-specific endocrine disruption, and the absence of overt placental inflammation or systemic maternal illness. Further studies using diverse MCMV and mouse strains, infection routes and doses, as well as detailed immunological profiling would help clarify these mechanistic differences.

This systematic comparison of multiple prenatal infections addresses a gap in our understanding of pregnancy complications. By examining diverse pathogens under standardised conditions, we have established experimental models that recapitulate some features of human prenatal infections. These models provide valuable tools for investigating the complex interactions between maternal immunity, placental function and fetal development. Our findings demonstrate that pregnancy outcomes are determined not simply by pathogen burden, but by the complex interplay between infection, inflammation and maternal adaptation.

### Limitation of the study

Despite sharing the latest common ancestor about 80 million years ago, mice and humans both have haemochorial placentae and so some of the processes that govern successful placentation in humans are shared with mice. Moreover, models of infection diseases are well established in mice. However, there are differences in reproductive biology between human and mouse models which limit the effectiveness of the comparisons made. The greatest disadvantage of mouse models is that much of organ development takes place after birth in mice, limiting the usefulness of the mouse as an informative model for later stages of human pregnancy. Also, trophoblast cells invade less deeply in mice than in humans ^22^. Because many pregnancy complications are rooted in defective placentation resulting from shallow trophoblast invasion, mice do not fully recapitulate adequately this aspect of placentation ^23^. Finally, NK-cell derived IFN-γ is the main cytokine that directs arterial remodelling in mice ^24^ whereas trophoblast drive the process in humans using several cytokienes and chemokines ^25^.

We focused immune profiling on the infections with the most overt maternal pathology ( *T. gondii* and VACV) and did not assess cytokine or immune cell responses in MCMV- and IAV-infected mice, which showed milder phenotypes under our conditions. This limits direct comparison across models and represents an area for future investigation.

Hormonal parameters were not assessed, and it remains possible that endocrine effects could influence pregnancy course and fetal outcomes.

## Ethics statement

Female C57BL/6 mice aged 7–10 weeks were maintained under specific pathogen-free conditions at the University of Cambridge in accordance with UK Home Office regulations (PPL PP2363781) approved by the Animal Welfare and Ethical Review bodies of the University Biomedical Services of the University of Cambridge, UK on 25/11/2019. The Animal Welfare and Ethical Review Body at the University of Cambridge Establishment Licence number, that is X81BD37B1. The University Welfare Assurance number which is F16-00007 (A5008-01).

To reduce pain, distress, or discomfort in mice, environmental enrichment was provided, such as nesting material, and hiding structures in the cages to promote natural behaviors and reduce stress. Housing conditions were optimised to allow for natural behaviors. Gentle handling techniques were used. Both researchers and technicians were properly trained in animal handling and pain assessment techniques. Mice were regularly observed for signs of distress like decreased activity, hunched posture, piloerection, or changes in eating/drinking patterns. Humane endpoints were implemented as indicated below. Mice received an administration of a category 1, 2 pathogen or control vehicle. Animals were closely monitored. The lowest dose of pathogens capable of inducing the clinical symptoms required for the study was used. The route of administration was selected to minimize distress while ensuring the clinical symptoms required for the study were induced. Pregnant mice were humanely killed before they gave birth at day 18.5 of gestation (day of vaginal plug discovery = 0.5 day of gestation). Weight loss was monitored and when it reached more than 15%, mice were killed. If the adverse effects of infection appeared greater than moderate, including if mice appear hunched, or display other non-transient severe clinical signs, e.g. piloerection, subdued behaviour, diarrhoea, they were killed by schedule 1 method. Any animal that lost 15% of its body weight gradually when compared to age-matched controls was killed.

## Data Availability

Figshare: Adverse maternal and fetal outcomes in mouse models of prenatal infections. https://doi.org/10.6084/m9.figshare.28058216 ^26^. This project contains the following underlying data: Animal weight changes (.xlsx format; daily weight records of experimental mice) Pathogen loads (.xlsx format; quantitative PCR and plaque assay data) Fetal and placental weight measurements (.xlsx format; measurements at gd 18.5) Vessel measurements from histological analyses (.xlsx format; quantification of uterine spiral artery parameters) Cytokine measurements (.xlsx format; Luminex assay data) Flow cytometry data (.xlsx format; immunophenotyping and cell count data) Data are available under CC BY 4.0 (Creative Commons Attribution 4.0 International). Figshare: Adverse maternal and fetal outcomes in mouse models of prenatal infections. https://doi.org/10.6084/m9.figshare.28058216 ^26^. This project contains the following extended data: Figure E1. Flow cytometry gating strategy for analysis of uterine leucocytes (.pdf format; detailing the flow cytometry gating strategy for uterine leucocytes) Figure E2. Pathogen load in tissues collected from in IAV and MCMV infected animals Data are available under CC BY 4.0 (Creative Commons Attribution 4.0 International). Figshare: ARRIVE checklist for ‘Adverse maternal and fetal outcomes in mouse models of prenatal infections’. https://doi.org/10.6084/m9.figshare.28058216 ^26^. Data are available under CC BY 4.0 (Creative Commons Attribution 4.0 International).
