# Supplementary material for: Adverse maternal and fetal outcomes in mouse models of prenatal infections
Source: Wellcome Open Res. 2025 Sep 9;10:142. Originally published 2025 Mar 18. [Version 2] doi: 10.12688/wellcomeopenres.23556.2 (PMC12481148; doi:10.12688/wellcomeopenres.23556.2)
Supplement: Peer review history

# Reviewer report: Reviewer response for version 2

**DOI:** 10.21956/wellcomeopenres.27434.r134140

**Published:** 2025-10-29

**Authors:** eunha kim

**Affiliations:** 1 Korea University College of Medicine, Seoul, Seoul, South Korea

Shmeleva *et al.* aimed to delineate pathogen-specific effects on both the maternal and fetal sides. The authors investigated four different pathogens and profiled the pathological phenotypes in mothers and fetuses. While the study comprehensively presents valuable observations, the following points require clarification and improvement:

1. The authors noted that there was no statistical significance in the *T. gondii* load in uterine tissues in figure 1D. However, the data appear to show a trend. It would be helpful if the authors provided their own interpretation by including specific statistical values between the groups. Please also indicate the statistical values for the VACV-infected groups.
2. Figure E2 is missing from the extended data.
3. It would strengthen the manuscript if the authors provided all data supporting the statements in the text. For example, the manuscript mentions that “litter sizes did not differ between infected and mock-infected groups” and that “while both toxoplasma and VACV were detected in the uterine tissue, including the trophoblast, there was no signal of either pathogen in the embryos,” but no corresponding data are shown.
4. The authors use both “toxoplasma” and *T. gondii* interchangeably. For clarity and consistency, please standardize the terminology throughout the manuscript. Note that *T. gondii* should be italicized.
5. The authors describe “increasing intensity from 5 dpi to 7 dpi,” but only a representative image is shown. Please quantify these signals to support the statement. Additionally, it would be helpful to indicate the decidual and placental regions in the representative figure with clear markings or annotations for the reader’s clarity.
6. The authors should explain why no non-pregnant sample was included for IAV infection.
7. In the manuscript, the authors mentioned “In uninfected controls, pregnant mice showed higher IL-1α levels compared to the non-pregnant group,” please include the statistical value directly on the graph.  

Is the work clearly and accurately presented and does it cite the current literature?

Yes

If applicable, is the statistical analysis and its interpretation appropriate?

Partly

Are all the source data underlying the results available to ensure full reproducibility?

Partly

Is the study design appropriate and is the work technically sound?

Yes

Are the conclusions drawn adequately supported by the results?

Yes

Are sufficient details of methods and analysis provided to allow replication by others?

Yes

Reviewer Expertise:

Immunology, cell biology, neuroscience

I confirm that I have read this submission and believe that I have an appropriate level of expertise to confirm that it is of an acceptable scientific standard, however I have significant reservations, as outlined above.

# Reviewer report: Reviewer response for version 2

**DOI:** 10.21956/wellcomeopenres.27434.r132858

**Published:** 2025-10-20

**Authors:** Rafaela José da Silva

**Affiliations:** 1 Universidade Federal de Uberlândia, Uberlândia, Brazil; 2 Biological Sciences, University of Pittsburgh Dietrich School of Arts and Sciences (Ringgold ID: 124478), Pittsburgh, Pennsylvania, USA

This manuscript presents a comparative analysis of four mouse models of prenatal infection, *Toxoplasma gondii*, *vaccinia virus, murine cytomegalovirus, *and *influenza A* virus, to investigate pathogen-specific effects on maternal and fetal health. The authors report marked differences in disease severity: *T. gondii *infection results in severe, pregnancy-specific pathology and maternal mortality, vaccinia virus induces prenatal morbidity, while *cytomegalovirus *and *influenza A *cause only mild and transient effects. Although none of the pathogens directly infected the fetuses, both *T. gondii* and vaccinia virus impaired uterine vascular remodeling and fetal growth. The study also shows that pregnancy alters local uterine immune responses, leading to reduced inflammatory cytokine production relative to non-pregnant controls.

 Overall, this is a well-designed and conceptually strong study that establishes a useful comparative framework for exploring pathogen-specific mechanisms underlying adverse pregnancy outcomes.

 To further strengthen the manuscript, I have several suggestions and questions for the authors to consider.

**Specific Comments:**

1. Please include the number of animals used in each experimental group to clarify sample size and strengthen the transparency of the study design.
2. It would be helpful to add details on the pathogen load quantification for MCMV in the Materials and Methods section.
3. The authors should clarify why pathogen localization data are not shown for the influenza virus and MCMV infections. Including these results, or explaining their absence, would improve completeness.
4. In the Results section, it would be informative to include representative images of uterine tissues infected with the other pathogens analyzed in the study, for consistency and visual comparison.
5. The authors mention that none of the pathogens were detected in embryonic tissues. Could the authors clarify whether pathogen load was assessed by PCR or another quantitative method? If not, incorporating such data would substantiate this conclusion.
6. It would also be useful to explain why cytokine responses were not measured in the MCMV-infected group, as this would allow more comprehensive comparison across infection models.
7. The statement “Previous studies have shown that IFN-γ production by NK cells can both protect against Toxoplasma infection and cause immunopathology during pregnancy” requires a supporting citation.

Is the work clearly and accurately presented and does it cite the current literature?

Yes

If applicable, is the statistical analysis and its interpretation appropriate?

Yes

Are all the source data underlying the results available to ensure full reproducibility?

Yes

Is the study design appropriate and is the work technically sound?

Yes

Are the conclusions drawn adequately supported by the results?

Partly

Are sufficient details of methods and analysis provided to allow replication by others?

Yes

Reviewer Expertise:

Immunology and Parasitology, with a focus on host–pathogen interactions in placental cells and their role in congenital infection and immune regulation at the maternal–fetal interface.

I confirm that I have read this submission and believe that I have an appropriate level of expertise to confirm that it is of an acceptable scientific standard.

# Reviewer report: Reviewer response for version 2

**DOI:** 10.21956/wellcomeopenres.27434.r132863

**Published:** 2025-10-11

**Authors:** Yusmaris Cariaco

**Affiliations:** 1 University of Ottawa, Ottawa, ON, Canada

This study systematically compares four mouse models of prenatal infection: Toxoplasma gondii, vaccinia virus, murine cytomegalovirus (MCMV), and influenza A virus, to understand pathogen-specific effects on pregnancy outcomes. The authors demonstrate that pregnancy profoundly modifies host responses to infection: T. gondii caused severe maternal morbidity and mortality due to uterine immunopathology; vaccinia virus led to fetal growth restriction and impaired vascular remodeling without direct fetal infection; MCMV produced mild maternal disease but reduced fetal and placental weights; while influenza A had minimal effects under the experimental conditions. Across models, none of the pathogens infected fetuses directly, indicating that maternal immune and vascular responses, rather than pathogen load, determine pregnancy outcomes. This study continues the group’s strong contributions to understanding placental immunology. I offer the following comments in the spirit of constructive feedback to enhance this study:

 Introduction / Methods:

 Could the authors clarify the rationale for using Vaccinia virus in this model? Was it intended to mimic MIA, or to simulate immune responses elicited by Vaccinia-based vaccines? Given that Vaccinia is not a TORCH pathogen and does not typically cause congenital infections in humans, further justification for its use in a prenatal infection model, ideally in the Introduction or Discussion, would strengthen the study.

 Please specify that the Toxoplasma gondii strain used is a type II strain, as this informs the reader of its expected virulence profile.

 The authors mention that sex was recorded but do not reference how it was determined, this would be helpful even if no sex-related differences were observed.

 There is no mention of the primers used for GRA2 DNA quantification nor PCR reaction specifics.

 The description of the tissue homogenization procedure is repetitive. The methods section could be streamlined by first describing common steps (“For T. gondii quantification, uterine tissues were collected at 7 days post-infection, homogenized in sterile PBS (0.4 ml) using a bead beater (4 cycles of 20 seconds)”) and then outlining experiment-specific details. For later procedures such as flow cytometry, the authors could reference this earlier description.

 The phrase “gd 9.5 implantation sites, together with uterine horns” is misleading, as implantation sites are anatomically located within the uterine horns. Please rephrase for clarity.

 The quantification method for MCMV is not described, nor is the tissue localization of MCMV and IAV. Even though these pathogens were undetectable, the methodology used to assess them should be mentioned.

 The authors mention, “Statistical analyses can alternatively be performed using R.” Were any of the analyses actually performed using R? If so, which ones? Please clarify or revise this statement.

 Results / Discussion:

 Please correct the use of toxoplasma to Toxoplasma throughout the manuscript.

 More scientific terminology should be used to describe clinical signs of morbidity. For example: “Oily coats” = Poor grooming, “Fur ruffling” = Piloerection, “Trembling” = Tremors, “Arched backs” = kyphosis or hunched posture.

 In addition, the discussion of morbidity based on the mortality curve is misleading, as the curve does not reflect clinical deterioration. The study by Bartley et al. (PMID: 16762097) provides a framework commonly used in murine models to assess morbidity caused by Apicomplexa parasites. The authors may wish to consider using this or a similar approach in future work, if the present dataset does not permit retrospective application.

 The authors should clarify whether pathological changes at Toxoplasma-infected implantation sites were first observed on 7 dpi or after 7 dpi. As currently written, the statement that “no notable differences were observed before 7 dpi,” followed by “dramatic changes at 7 and 8 dpi,” is logically inconsistent. Figures show only 8dpi vs non infected mice in which clear macroscopic differences are noted.

 Since the study focuses on maternal health and pregnancy outcomes, is there reason for investigating ovarian dissemination of Vaccinia virus? Considering that the authors cite ovary-related viral dissemination mechanisms for MCMV in the discussion, it may be more relevant to explore this aspect for that virus instead.

 Please correct “Intravenously” to “intravenous” in the description of MCMV in Figure 1A.

 Figures E2A and E2B are referenced but do not appear in the supplementary materials.

 Given that mortality of Toxoplasma-infected mice occurs between 5–8 dpi, the authors could have considered establishing an additional mouse cohort or reducing the infectious inoculum to avoid maternal mortality.

 In Figure 2C, the confocal images are shown at such low magnification and contrast that it is difficult to identify decidua and trophoblast regions. Higher-quality images or annotations would improve clarity.

 It is unclear why the authors did not measure type I interferons, which are more appropriate markers for acute viral infections than type II interferons. Since the study includes three different viral infections, this seems particularly relevant. Is this related to IFN-g production by dNK cells?

 Although the authors later acknowledge this limitation, beginning the discussion by calling this a comparative study of four models of prenatal infection is somewhat inaccurate. Technical and biological limitations prevented consistent parameter comparisons across all models. With additional data, this study might have been more impactful if split into more focused experiments.

 Figure 2B does not appear to show clear evidence of vascular congestion following Toxoplasma infection, contrary to what is stated in the Discussion.

 References cited seem limited. Authors could enrich discussion section with more articles supporting or contradicting their observations. For example, this is -if not the first- one of the few articles clearly showing spiral arteries remodeling failure in T. gondii infection. Previous studies have mentioned arteries dilation and congestion (PMID: 28603517, PMID: 18838076),  but few a proper SA remodeling analysis (PMID: 18838076).

Is the work clearly and accurately presented and does it cite the current literature?

Yes

If applicable, is the statistical analysis and its interpretation appropriate?

Yes

Are all the source data underlying the results available to ensure full reproducibility?

Partly

Is the study design appropriate and is the work technically sound?

Yes

Are the conclusions drawn adequately supported by the results?

Yes

Are sufficient details of methods and analysis provided to allow replication by others?

Partly

Reviewer Expertise:

Placenta biology, pregnancy-associated diseases, Plasmodium and Toxoplasma infections

I confirm that I have read this submission and believe that I have an appropriate level of expertise to confirm that it is of an acceptable scientific standard, however I have significant reservations, as outlined above.

# Reviewer report: Reviewer response for version 2

**DOI:** 10.21956/wellcomeopenres.27434.r132041

**Published:** 2025-09-29

**Authors:** Stipan Jonjic

**Affiliations:** 1 University of Rijeka, Rijeka, Croatia

The authors have submitted a revised manuscript that adequately addresses the main issues raised during the initial review. The revisions include clearer explanations and an improved structure, enhancing the text’s accessibility and strengthening the overall narrative. The responses to the reviewers’ comments were thorough, and the authors made noticeable efforts to integrate these clarifications into the manuscript. The expanded discussion offers a better context for the findings and emphasizes the broader relevance of the study.

 While the textual improvements increase clarity and flow, no additional experiments were conducted. Nonetheless, the current version is a clear improvement over the original submission, addressing the major concerns raised. The manuscript is now clearer, more comprehensive, and better suited for the field’s readership. I find the revised paper acceptable in its present form, while recommending that the authors consider additional experimental approaches in future work to strengthen and extend their findings.

Is the work clearly and accurately presented and does it cite the current literature?

Partly

If applicable, is the statistical analysis and its interpretation appropriate?

Partly

Are all the source data underlying the results available to ensure full reproducibility?

Yes

Is the study design appropriate and is the work technically sound?

Partly

Are the conclusions drawn adequately supported by the results?

Partly

Are sufficient details of methods and analysis provided to allow replication by others?

Partly

Reviewer Expertise:

NA

I confirm that I have read this submission and believe that I have an appropriate level of expertise to confirm that it is of an acceptable scientific standard.

# Reviewer report: Reviewer response for version 2

**DOI:** 10.21956/wellcomeopenres.27434.r132040

**Published:** 2025-09-17

**Authors:** Stella Liong

**Affiliations:** 1 RMIT University, Bundoora, Australia

The authors have addressed my feedback and concerns sufficiently and I have no further comments.

Is the work clearly and accurately presented and does it cite the current literature?

Partly

If applicable, is the statistical analysis and its interpretation appropriate?

Yes

Are all the source data underlying the results available to ensure full reproducibility?

Yes

Is the study design appropriate and is the work technically sound?

Yes

Are the conclusions drawn adequately supported by the results?

Yes

Are sufficient details of methods and analysis provided to allow replication by others?

Yes

Reviewer Expertise:

NA

I confirm that I have read this submission and believe that I have an appropriate level of expertise to confirm that it is of an acceptable scientific standard.

# Reviewer report: Reviewer response for version 1

**DOI:** 10.21956/wellcomeopenres.25987.r120966

**Published:** 2025-05-08

**Authors:** Stella Liong

**Affiliations:** 1 RMIT University, Bundoora, Australia

Shmeleva et al assessed the effect of *T. gondii,* vaccinia, MCMV and IAV infection during pregnancy. The authors identified *T.gondii* infection was associated with high disease severity and pregnancy loss due to vascular pathology driven by *T. gondii* infection. I have some comments and questions regarding the study:

 1) Why was MCMV inoculation performed at GD6.5 whereas the other infection models were performed at GD4.5?

 2) Were there any changes in litter sizes between infected and mock-infected groups?

 3) No spiral artery remodelling data (Fig 2B) shown for IAV infection. There is some literature to suggest that IAV infection also impacts vascular function in pregnancy. Was this not assessed or was there no change with IAV infection?

 4) The authors argue that IAV infection in pregnant mice does not recapitulate disease severity seen in human pregnancies with the authors citing 1 study. However there has been other many preclinical studies which have shown IAV infection can promote severe disease during pregnancy compared to non-pregnant mice and should be discussed.

 5) I believe there is a typo on the last line of the limitation of the study section of the discussion "whereas trophoblast drive the process in mice using...". I believe it should be humans rather than mice, as the study cited used human cells.

 6) Methods (Pathogen load quantification) - only T.gondii and VACV quantification methods were provided. IAV quantification metholody is missing despite authors stating viral titres were measured (data not shown). Please add method for IAV.

 7) Methods (Animal procedures) - The authors stated that sex was noted from GD18.5 foetuses, was any sex analysis performed?

Is the work clearly and accurately presented and does it cite the current literature?

Partly

If applicable, is the statistical analysis and its interpretation appropriate?

Yes

Are all the source data underlying the results available to ensure full reproducibility?

Yes

Is the study design appropriate and is the work technically sound?

Yes

Are the conclusions drawn adequately supported by the results?

Yes

Are sufficient details of methods and analysis provided to allow replication by others?

Yes

Reviewer Expertise:

immunology, cardiovascular, pregnancy, influenza infection

I confirm that I have read this submission and believe that I have an appropriate level of expertise to confirm that it is of an acceptable scientific standard, however I have significant reservations, as outlined above.

# Response

**Published:** 2025-09-01

**Authors:** Francesco Colucci

1) Why was MCMV inoculation performed at GD6.5 whereas the other infection models were performed at GD4.5?

 R-cThank you for bringing this up and apologies for not clarify this point. We decided the time of infection for each pathogen based on relative peak immune responses to the specific pathogen. Guided by that, infection time points were set to make all immune response peak coincidentally with the time in gestation when vascular remodelling occurs at gd 9.5–11.5. Because the response to T. gondii, VACV and IAV peaks at 5–8 dpi, while the response to MCMV peaks earlier, we set gd 4.5 for the first three pathogens and gd 6.5 for the latter. By infecting at these time points, we synchronised the infection models with the pregnancy-specific tissue remodelling, despite differences in pathogens' replication dynamics and tissue tropism. We have now incorporated this explanation in the Methods section, under “Animal procedures,” immediately after the sentence specifying infection time points.  

 2) Were there any changes in litter sizes between infected and mock-infected groups?

 R-Litter sizes were recorded, and we did not observe any differences between infected and mock-infected groups. A sentence to this effect has been added to the Results section following the description of fetal and placental weights.  

 3) No spiral artery remodelling data (Fig 2B) shown for IAV infection.

 There is some literature to suggest that IAV infection also impacts vascular function in pregnancy. Was this not assessed or was there no change with IAV infection?

 Spiral artery remodelling was not assessed in the IAV-infected group in our study. Our primary aim was to compare local immune and vascular responses across models that showed clear pathological changes. In our experimental conditions, IAV-infected pregnant mice exhibited minimal morbidity and no apparent pregnancy-related pathology. Given the labour–intensive nature of histological analysis, we did not prioritise vascular assessment in this model. We now clarify this point in the *Methods* section.  

 4) The authors argue that IAV infection in pregnant mice does not recapitulate disease severity seen in human pregnancies with the authors citing 1 study. However there has been other many preclinical studies which have shown IAV infection can promote severe disease during pregnancy compared to non-pregnant mice and should be discussed.

 Thank you for pointing this out and apologise for our oversight. Numerous murine studies have indeed reported greater morbidity or mortality in pregnant versus non-pregnant mice after IAV challenge, particularly when BALB/c mice, higher-dose or highly pathogenic viral strains, and mid-to-late gestational infections were used (Littauer et al., 2017, PMID: 29176767; Lauzon-Joset et al., 2019, PMID: 30576502; Creisher et al., 2024, PMID: PMID: 38190129). However, recent work in C57BL/6 mice has shown pregnancy-associated protection linked to enhanced antiviral defence in the upper airways (Chronopoulos et al., 2024, PMID: 39331716). We have used C57BL/6 dams, an early-gestation infection (gd 4.5), and a low, sub-lethal dose of the PR8 H1N1 strain. Under these specific conditions the infected pregnant mice displayed minimal morbidity, consistent with Chronopoulos et al. We have now expanded the Discussion to acknowledge the broader literature and to emphasise that IAV outcome in pregnancy is highly contingent on mouse background, viral strain and dose and gestational timing.  

 5) I believe there is a typo on the last line of the limitation of the study section of the discussion "whereas trophoblast drive the process in mice using...". I believe it should be humans rather than mice, as the study cited used human cells.

 Thank you for spotting this error. Indeed, the sentence should read “in humans” rather than “in mice”, as the cited study (Li et al., 2024) used human extravillous trophoblasts and uterine NK cells. This has now been corrected in the *Limitations* section.

   6) Methods (Pathogen load quantification) - only T.gondii and VACV quantification methods were provided. IAV quantification metholody is missing despite authors stating viral titres were measured (data not shown). Please add method for IAV. Thank you for highlighting this omission and apologies for our mistake. We have now added a description of the viral quantification method for IAV to the Pathogen load quantification section of the Methods.  

 7) Methods (Animal procedures) - The authors stated that sex was noted from GD18.5 foetuses, was any sex analysis performed?

 Yes, fetal sex was recorded at gd 18.5; however, no differences were observed between male and female fetuses in any of the outcome measures assessed. Therefore, sex-specific analyses were not pursued further. We now clarify this in the Methods.

# Reviewer report: Reviewer response for version 1

**DOI:** 10.21956/wellcomeopenres.25987.r120970

**Published:** 2025-04-25

**Authors:** Stipan Jonjic, Vanda Juranić Lisnić

**Affiliations:** 1 University of Rijeka, Rijeka, Croatia; 2 Faculty of Medicine, University of Rijeka, Croatia, Croatia

The authors conducted a comparative analysis of perinatal infections using four pathogens: Toxoplasma gondii, vaccinia virus, cytomegalovirus (CMV), and influenza virus. The study aimed to investigate systemic and local immune responses during pregnancy, as well as the impact on vascular remodeling, placentation, and fetal development.

 Consistent with previous reports, infection with T. gondii resulted in severe pregnancy-specific pathology, including maternal mortality. In contrast, infections with vaccinia virus, CMV, and influenza virus were associated with milder—though distinct—effects. The authors hypothesize that perinatal morbidity following T. gondii infection may be driven by immunopathological mechanisms, whereas the morbidity observed with vaccinia virus could be attributed to direct tissue infection. Notably, both T. gondii and vaccinia virus infections were shown to alter uterine vascular remodeling and impair fetal growth.

 Understanding pathogen-specific effects on pregnancy is undoubtedly critical for the development of targeted therapeutic interventions. However, the rationale behind the selection of these particular pathogens for direct comparison is not entirely clear. Given their divergent routes of infection, biological characteristics, and pathogenic mechanisms, drawing overarching conclusions from such a heterogeneous group is not fully justified. Thus, although the study presents findings that may inform more focused investigations into individual pathogen- host interactions during pregnancy, I cannot support this manuscript in this version. A major revision is needed to comply with the list of questions mentioned below.

 Below are several specific comments for the authors' consideration:

1. The introduction lacks a short overview of the diseases caused by the investigated pathogens. It is also not clear why particular time points were chosen for infection.
2. Incomplete immune profiling: Although four different pathogens were studied, cytokine and immune response analyses are lacking for MCMV-infected mice. Furthermore, immune cell responses were only assessed for T. gondii and vaccinia virus, while data for MCMV and influenza A virus (IAV) are missing. Including these data would significantly strengthen the comparative aspect of the study. However, even that is questionable because of different routes of infection.
3. Inconsistent infection time points: It is unclear why different gestational time points were used for infections with different pathogens. Specifically, infections with T. gondii, VACV, and IAV were performed at 4.5 days post coitum (dpc), while MCMV infection was carried out at 6.5 dpc. The authors should clarify whether there is a specific biological or methodological rationale for this discrepancy.
4. Lack of evidence for absence of fetal infection: The authors state that none of the tested pathogens caused direct fetal infection, but no experimental evidence is provided to support this claim. It appears that this assumption may be based on previous findings, which should be clearly cited to support the statement. On the other hand, for MCMV it is well established that unlike HCMV, MCMV does not cross the placental barrier, and this important distinction should be taken into account when comparing experimental models.
5. VACV effects on pregnancy not addressed adequately: The authors report minimal impact of VACV on pregnancy outcomes and fetal mortality. However, prior studies, such as Benning et al. (J Virol, 2004), have demonstrated significant pathogenic effects, including dominant infection of the ovaries ( https://pmc.ncbi.nlm.nih.gov/articles/PMC353726/). VACV infection at early stage, may compromise hormonal balance and fetal development due to the overwhelming infection of ovaries. Did the authors assess hormone levels to evaluate this potential mechanism?
6. Cytokine profiling: Given the fact that the uterine is highly vascularized, how did the researchers ensure that the measured cytokines simply do not reflect cytokines in the plasma? In light of that, the author's conclusion that "the tissue of pregnant mice displayed a much-reduced rise in TNF-α, IFN-γ, and CXCL10, and no rise in IL-6, suggesting that pregnancy blunts the local inflammatory response” is not convincing.
7. Insufficient integration of MCMV literature: The discussion lacks key references from the MCMV field. In light of previous studies reporting detrimental effects of MCMV on pregnancy, the authors should provide a critical analysis of why no such effects were observed in their study.

Additional Comments:

 Figure 1A:

 For improved clarity and comparison, it would be helpful if all y-axes were standardized to the same maximum value (e.g., 200), where applicable.

 Figure 1D:

 Interestingly, despite the severe clinical signs caused by T. gondii infection in pregnant mice compared to non-pregnant mice, there was no significant difference in pathogen load in uterine tissue between the two of the T. gondii infected groups. It is unclear what statistics were used and what the dashed line means. Additionally, the analysis of pathogen genome copy number in a control organ—such as the lungs, as was performed for VACV—is lacking. Quantification of MCMV and IAV viral loads in uterine tissue is also missing and would be important to include for completeness. This experiment should be repeated with a bit larger number of animals.

 Figure 2B:

 The vascular remodeling analysis would be significantly enhanced by including representative histological images (e.g., hematoxylin and eosin staining) to visually support the quantitative data.

 Figure 3:

 There appears to be an inconsistency in Fig. 3A, where the IAV-infected non-pregnant group is missing, although it is shown in Fig. 3B. Furthermore, the legend indicates that filled circles represent non-pregnant mice, while in the graph they are shown as empty circles. This discrepancy should be clarified and corrected.

 Figure 4:

 Why was the immune response not analyzed in MCMV- and IAV-infected mice, as it was for the other pathogens? In addition, the second graph contains axis labels "DP" and "DN," which are not defined in the figure legend. These abbreviations should be clearly explained.

Is the work clearly and accurately presented and does it cite the current literature?

Partly

If applicable, is the statistical analysis and its interpretation appropriate?

Partly

Are all the source data underlying the results available to ensure full reproducibility?

Yes

Is the study design appropriate and is the work technically sound?

Partly

Are the conclusions drawn adequately supported by the results?

Partly

Are sufficient details of methods and analysis provided to allow replication by others?

Partly

Reviewer Expertise:

Viral immunology and pathogenesis, congenital infections

We confirm that we have read this submission and believe that we have an appropriate level of expertise to state that we do not consider it to be of an acceptable scientific standard, for reasons outlined above.

## References

[1] : Vaccinia virus infection during murine pregnancy: a new pathogenesis model for vaccinia fetalis. *J Virol* .2004;78(6) : 10.1128/jvi.78.6.3133-3139.2004 3133-9 10.1128/jvi.78.6.3133-3139.2004 14990732 PMC353726

# Response

**Published:** 2025-09-01

**Authors:** Francesco Colucci

1. The introduction lacks a short overview of the diseases caused by the investigated pathogens. It is also not clear why particular time points were chosen for infection.

We have revised the Background section and included a short overview of the clinical relevance and disease associations of each pathogen included in the study. We have also clarified the choice of infection time points in the Methods (under Animal procedures) to explain how they were selected to synchronise peak immune responses with mid-gestation (gd 9.5–11.5), when vascular remodelling is actively occurring - see also our response to Reviewer 1, Comment 1.  

1. Incomplete immune profiling: Although four different pathogens were studied, cytokine and immune response analyses are lacking for MCMV-infected mice. Furthermore, immune cell responses were only assessed for T. gondii and vaccinia virus, while data for MCMV and influenza A virus (IAV) are missing. Including these data would significantly strengthen the comparative aspect of the study. However, even that is questionable because of different routes of infection.

We agree with the reviewer that a full immune profiling across all four models would have strengthened the comparative aspect of the work. However, we focused on immunological analyses of responses to T. gondii and VACV, because these two pathogens showed the most pronounced maternal morbidity and uterine pathology. We attempted to characterise in greater depth the models with the clearest pregnancy-specific effects, rather than applying uniform profiling across all infections - which, we agree with the Reviewers, would have been ideal, but budget constraints were also considered. For MCMV and IAV, which caused only mild or no maternal disease and limited histopathological changes under our experimental conditions, we did not pursue immune cell phenotyping or cytokine profiling in this initial comparison. We now acknowledge this limitation in the Limitation section.   We accept that differences in pathogen tropism, virulence, and route of infection may confound direct immunological comparisons. Nonetheless, we believe that these models together provide a useful framework for dissecting how distinct pathogens interact with maternal adaptations to pregnancy.  

1. Inconsistent infection time points: It is unclear why different gestational time points were used for infections with different pathogens. Specifically, infections with T. gondii, VACV, and IAV were performed at 4.5 days post coitum (dpc), while MCMV infection was carried out at 6.5 dpc. The authors should clarify whether there is a specific biological or methodological rationale for this discrepancy.

Thank you for bringing this up and apologies for not clarify this point. We decided the time of infection for each pathogen based on relative peak immune responses to the specific pathogen. Guided by that, infection time points were set to make all immune response peak coincidentally with the time in gestation when vascular remodelling occurs at gd 9.5–11.5. Because the response to T. gondii, VACV and IAV peaks at 5–8 dpi, while the response to MCMV peaks earlier, we set gd 4.5 for the first three pathogens and gd 6.5 for the latter. By infecting at these time points, we synchronised the infection models with the pregnancy-specific tissue remodelling, despite differences in pathogens' replication dynamics and tissue tropism. We have now incorporated this explanation in the Methods section, under “Animal procedures,” immediately after the sentence specifying infection time points. See also response to Reviewer 1, Comment #1.  

1. Lack of evidence for absence of fetal infection: The authors state that none of the tested pathogens caused direct fetal infection, but no experimental evidence is provided to support this claim. It appears that this assumption may be based on previous findings, which should be clearly cited to support the statement. On the other hand, for MCMV it is well established that unlike HCMV, MCMV does not cross the placental barrier, and this important distinction should be taken into account when comparing experimental models.

The conclusion that none of the pathogens caused direct fetal infection is supported by several lines of experimental evidence. For T. gondii and VACV, we performed both pathogen load quantification (qPCR or plaque assay) and localisation imaging on whole implantation sites. Fluorescent-tagged T. gondii and VACV were consistently detected in maternal decidua and placental regions, but not in embryonic tissues (Figure 2C). For MCMV and IAV, plaque assays were performed on homogenates of whole implantation sites, and no infectious virus was detected. As the reviewer has noted, in the case of MCMV, this is also consistent with established literature showing that MCMV does not cross the placenta in immunocompetent mice, unlike HCMV.  

1. VACV effects on pregnancy not addressed adequately: The authors report minimal impact of VACV on pregnancy outcomes and fetal mortality. However, prior studies, such as Benning et al. (J Virol, 2004), have demonstrated significant pathogenic effects, including dominant infection of the ovaries ( https://pmc.ncbi.nlm.nih.gov/articles/PMC353726/). VACV infection at early stage, may compromise hormonal balance and fetal development due to the overwhelming infection of ovaries. Did the authors assess hormone levels to evaluate this potential mechanism?

We agree that prior studies, including Benning et al. (2004), have shown that VACV infection can severely impact pregnancy, including ovarian tropism and fetal loss. However, this discrepancy with our results is likely due to key differences in experimental design. Benning et al. used BALB/c mice, a strain known to be highly susceptible to WR strain vaccinia virus. Also, Benning et al. administered 1 × 10⁶ PFU intranasally at gd 7, which led to systemic viral dissemination and high maternal mortality. In contrast, we used C57BL/6 mice, which are more resistant to VACV-WR infection, and a 1000-fold lower dose (2 × 10³ PFU at gd 4.5), closer to the threshold for sublethal respiratory infection.   We did assess ovaries in infected animals. In two independent experiments, plaque assays on ovary homogenates at 7 dpi were uniformly negative, with no infectious virus detected (detection limit ≈ 50 PFU/g). Given this result, we did not pursue hormone analysis, as direct ovarian involvement appeared unlikely.   We now clarify this in the Results, discuss the relevance of dose, strain, and gestational timing in the Discussion, and have added a statement in the Limitations noting that hormonal parameters were not assessed and that endocrine effects cannot be excluded.  

1. Cytokine profiling: Given the fact that the uterine is highly vascularized, how did the researchers ensure that the measured cytokines simply do not reflect cytokines in the plasma? In light of that, the author's conclusion that "the tissue of pregnant mice displayed a much-reduced rise in TNF-α, IFN-γ, and CXCL10, and no rise in IL-6, suggesting that pregnancy blunts the local inflammatory response” is not convincing.

  Thank you for this critical observation. We agree that we may not be able to definitely exclude that, without perfusion, some plasma-derived cytokines may contribute to the measured levels. However, several aspects of our findings support genuine tissue-specific regulation:   First, cytokine profiles in tissue and plasma were not identical. For example, IL-6 was elevated in uterine tissue of VACV-infected mice but not in plasma, while IL-1α showed opposite trends in tissue versus plasma between pregnant and non-pregnant animals (Figure 3). This divergence argues against passive diffusion as the sole source of signal.   Second, uterine tissue samples were homogenised mechanically using bead-beating, which yields intracellular and matrix-associated cytokines, not just vascular content.   Third, the difference in tissue cytokine levels between pregnant and non-pregnant mice — despite no difference in plasma levels — supports the conclusion that pregnancy modulates the local inflammatory response.   Thus, our current data may well reflect true tissue-level regulation and could justify our conclusion that pregnancy blunts local inflammation.  

1. Insufficient integration of MCMV literature: The discussion lacks key references from the MCMV field. In light of previous studies reporting detrimental effects of MCMV on pregnancy, the authors should provide a critical analysis of why no such effects were observed in their study.

Thank you for pointing this out. We have now expanded the MCMV section of the Discussion to place our findings in the wider literature context and to explain why severe outcomes reported by others were not replicated here.     Additional Comments: Figure 1A: For improved clarity and comparison, it would be helpful if all y-axes were standardized to the same maximum value (e.g., 200), where applicable. We agree that standardazed y-axes are a more rigorous way of presenting data and we would have preferred to do that. However, the magnitude and direction of weight changes varied substantially across models, and applying a uniform y-axis would have compressed differences, reducing the clarity of the data. Therefore, to preserve readability of the data, we did opt for axis scaling that was tailored to each experimental condition.   Figure 1D: Interestingly, despite the severe clinical signs caused by T. gondii infection in pregnant mice compared to non-pregnant mice, there was no significant difference in pathogen load in uterine tissue between the two of the T. gondii infected groups. It is unclear what statistics were used and what the dashed line means. Additionally, the analysis of pathogen genome copy number in a control organ—such as the lungs, as was performed for VACV—is lacking. Quantification of MCMV and IAV viral loads in uterine tissue is also missing and would be important to include for completeness. This experiment should be repeated with a bit larger number of animals. Thank you for these detailed observations.

- Statistical comparisons in Figure 1D were performed using the Mann–Whitney U test, as described in the Statistical analysis section of the Methods:

‘To verify that the data met the assumptions underlying the statistical tests, we initially assessed the normality of each dataset using the Shapiro-Wilk test and by visual inspection of Q-Q plots. Non-parametric Mann-Whitney U tests were used for comparisons between two experimental groups. For longitudinal analyses of weight changes, two-way repeated measures analysis of variance (ANOVA) was applied, with time and experimental condition as factors. P values < 0.05 were considered statistically significant; *p < 0.05, **p < 0.01, ***p < 0.001, ****p < 0.0001. Effect sizes with corresponding confidence intervals were not computed for the analyses presented in this study.’  

- The dashed line represents the assay’s lower limit of detection - this has now been clarified in the figure legend.

 

- Lung viral titres for VACV were assessed by plaque assay and are shown in Figure 1D, as a direct comparison between pregnant and non-pregnant mice. Regarding the use of genome copy number, this was not performed for VACV, as the field standard is plaque assay (PFU), which specifically quantifies infectious particles. This is particularly relevant for VACV, where a high proportion of virions may be non-infectious due to defective wrapping or incomplete assembly. PFU thus provides a more biologically meaningful readout of active infection and is the gold standard for quantification in orthopoxvirus research. We therefore did not consider qPCR-based genome quantification necessary or appropriate in this context.

 

- Uterine viral loads were quantified for both MCMV and IAV, and these data are now provided in Supplementary Figure 2 (Figure E2).

For MCMV, pregnant mice were infected intravenously at gd 6.5, and uterine tissues were collected at 3 dpi alongside spleen, liver, and blood. Plaque assays showed robust viral titres in the uterus, confirming replication in obtained tissues. MCMV viral loads were assessed only for pregnant animals. For IAV, pregnant and non-pregnant mice were infected intranasally with 10³ PFU (PR8 strain), and tissues were collected at 7 dpi. Plaque assays demonstrated viral titres in the lungs, while no infectious virus was detected in uterine tissue. We thank the reviewer for prompting inclusion of these data, which are now referenced in the Results and included in the supplementary material for transparency and completeness.  

- While we agree that larger group sizes would always enhance statistical power, the pathogen load data presented are based on at least two independent experiments with consistent results. As the reviewer notes, the conclusion that T. gondii causes severe disease without increased tissue load remains clear from the existing data. Therefore, we respectfully maintain the current experimental scope.

  Figure 2B: The vascular remodeling analysis would be significantly enhanced by including representative histological images (e.g., hematoxylin and eosin staining) to visually support the quantitative data. We thank the reviewer for this suggestion and agree that a " *picture is worth a thousand words*", but in some circumstances this may be misleading. Vascular remodelling analysis was performed using a well-established and peer-reviewed protocol (Kieckbusch et al., J Vis Exp 2015), which involves quantitative measurement of spiral artery morphology across multiple vessels and implantation sites. As described in the Methods, we assessed the five largest, roundest vessels in three implantation sites per animal, performing 15 measurements per animal to calculate mean lumen area, vessel area, and vessel-to-lumen ratios. Because the analysis is based on cumulative quantitative data from carefully selected vessels, no single histological image would adequately represent the data distribution or vessel selection criteria. Moreover, variability in sectioning angle and vessel orientation makes representative imaging prone to misinterpretation. For these reasons, and to avoid over-reliance on qualitative impressions, we have not included individual H&E images. We believe the robust quantitative approach used provides the most accurate and reproducible assessment of vascular remodelling.   Figure 3: There appears to be an inconsistency in Fig. 3A, where the IAV-infected non-pregnant group is missing, although it is shown in Fig. 3B. Furthermore, the legend indicates that filled circles represent non-pregnant mice, while in the graph they are shown as empty circles. This discrepancy should be clarified and corrected. We thank the reviewer for this careful observation. Cytokine and chemokine levels were not measured in uterine tissue from IAV-infected animals; therefore, the IAV groups do not appear in Figure 3A. As discussed in the Results and Discussion, IAV caused minimal uterine pathology in our model, and viral titres in the uterus were undetectable at 7 dpi (Figure E2A). Instead, cytokine profiling in this model was focused on plasma to assess systemic immune responses (Figure 3B). Regarding the figure symbols, this was a typo in the figure legend, and we thank the reviewer for spotting it. The legend has now been corrected, so that filled circles represent pregnant animals and empty circles represent non-pregnant animals, consistent with the other figures.   Figure 4: Why was the immune response not analyzed in MCMV- and IAV-infected mice, as it was for the other pathogens? In addition, the second graph contains axis labels "DP" and "DN," which are not defined in the figure legend. These abbreviations should be clearly explained.

- We agree with the reviewer that a full immune profiling across all four models would have strengthened the comparative aspect of the work. However, we focused on immunological analyses of responses to T. gondii and VACV, because these two pathogens showed the most pronounced maternal morbidity and uterine pathology. We attempted to characterise in greater depth the models with the clearest pregnancy-specific effects, rather than applying uniform profiling across all infections - which, we agree with the Reviewers, would have been ideal, but budget constraints were also considered. For MCMV and IAV, which caused only mild or no maternal disease and limited histopathological changes under our experimental conditions, we did not pursue immune cell phenotyping or cytokine profiling in this initial comparison. We now acknowledge this limitation in the Limitation section.

We accept that differences in pathogen tropism, virulence, and route of infection may confound direct immunological comparisons. Nonetheless, we believe that these models together provide a useful framework for dissecting how distinct pathogens interact with maternal adaptations to pregnancy.

- Regarding the figure, thank you for spotting our omission. We have updated the legend for Figure 4 to define “DP” as double-positive (CD4+CD8+) and “DN” as double-negative (CD4-CD8-) T cells. We appreciate the reviewer’s attention to clarity and detail in presentation.
